# Newly recognized cerebral infarctions on postmortem imaging: a report of three cases with systemic infectious disease

**DOI:** 10.1186/s12880-016-0174-4

**Published:** 2017-01-10

**Authors:** Sakon Noriki, Kazuyuki Kinoshita, Kunihiro Inai, Toyohiko Sakai, Hirohiko Kimura, Takahiro Yamauchi, Masayuki Iwano, Hironobu Naiki

**Affiliations:** 1 Division of Tumor Pathology, Department of Pathological Sciences, School of Medical Sciences, University of Fukui, 23-3 Shimoaizuki, Matsuoka Eiheiji-cho, Yoshida-gun, 910-1193 Fukui, Japan; 2 Division of Radiology, Department of Radiology and Laboratory Medicine, School of Medical Sciences, University of Fukui, 23-3 Shimoaizuki, Matsuoka Eiheiji-cho, Yoshida-gun, 910-1193 Fukui, Japan; 3 Division of Molecular Pathology, Department of Pathological Sciences, School of Medical Sciences, University of Fukui, Fukui, Japan; 4 Autopsy Imaging Center, School of Medical Sciences, University of Fukui, Fukui, Japan; 5 Division of Hematology and Oncology, Faculty of Medical Sciences, University of Fukui, 23-3 Shimoaizuki, Matsuoka Eiheiji-cho, Yoshida-gun, 910-1193 Fukui, Japan; 6 Division of Nephrology, Department of General Medicine, University of Fukui, 23-3 Shimoaizuki, Matsuoka Eiheiji-cho, Yoshida-gun, 910-1193 Fukui, Japan

**Keywords:** Case report, Postmortem imaging, Cerebral infarction, Infection, Cause of death, Autopsy, Pathology

## Abstract

**Background:**

Postmortem imaging (PMI) refers to the imaging of cadavers by computed tomography (CT) and/or magnetic resonance imaging (MRI). Three cases of cerebral infarctions that were not found during life but were newly recognized on PMI and were associated with severe systemic infections are presented.

**Case presentations:**

An 81-year-old woman with a pacemaker and slightly impaired liver function presented with fever. Imaging suggested interstitial pneumonia and an iliopsoas abscess, and blood tests showed liver dysfunction and disseminated intravascular coagulation (DIC). Despite three-agent combined therapy for tuberculosis, she died 32 days after hospitalization. PMI showed multiple fresh cerebral and cerebellar infarctions and diffuse ground-glass shadows in bilateral lungs. On autopsy, the diagnosis of miliary tuberculosis was made, and non-bacterial thrombotic endocarditis that involved the aortic valve may have caused the cerebral infarctions.

A 74-year-old man on steroid therapy for systemic lupus erythematosus presented with severe anemia, melena with no obvious source, and DIC. Imaging suggested intestinal perforation. The patient was treated with antibiotics and drainage of ascites. However, he developed adult respiratory distress syndrome, worsening DIC, and renal dysfunction and died 2 months after admission. PMI showed infiltrative lung shadow, ascites, an abdominal aortic aneurysm, a wide infarction in the right parietal lobe, and multiple new cerebral infarctions. Autopsy examination showed purulent ascites, diffuse peritonitis, invasive bronchopulmonary aspergillosis, and non-bacterial thrombotic endocarditis that likely caused the cerebral infarctions.

A 65-year-old man with an old pontine infarction presented with a fever and neutropenia. Despite appropriate treatment, his fever persisted. CT showed bilateral upper lobe pneumonia, pain appeared in both femoral regions, and intramuscular abscesses of both shoulders developed. His pneumonia worsened, his level of consciousness decreased, right hemiplegia developed, and he died. PMI showed a newly diagnosed cerebral infarction in the left parietal lobe. The autopsy revealed bilateral bronchopneumonia, right-sided pleuritis with effusion, an intramuscular abscess in the right thigh, and fresh multiple organ infarctions. Systemic fibrin thrombosis and DIC were also found. Postmortem cultures showed *E. coli* and *Burkholderia cepacia*.

**Conclusion:**

Cerebral infarction that is newly recognized on PMI might suggest the presence of severe systemic infection.

**Electronic supplementary material:**

The online version of this article (doi:10.1186/s12880-016-0174-4) contains supplementary material, which is available to authorized users.

## Background

Cadavers can be evaluated using diagnostic imaging, which comprises one aspect of medical assessment at the time of death. Imaging of cadavers has also been referred to as postmortem imaging (PMI). However, this procedure is variously described as virtopsy in Switzerland [1], virtual autopsy in France [2], radio-autopsy in Germany [3], and autopsy imaging (Ai) in Japan [4]. Although the descriptions and concept of PMI in these countries differ somewhat, all involve analysis of a cadaver by computed tomography (CT) and/or magnetic resonance imaging (MRI) to acquire postmortem medical information.

PMI is a useful diagnostic tool for a forensic case [5] that has no antemortem medical information. However, PMI is often performed in hospital deaths. On the other hand, the rate of the hospital autopsies has been decreasing worldwide in recent years, because the hospital autopsy requires consent in most countries. Furthermore, the brain examination rate of the hospital autopsy is only 20% at our hospital, because another consent is required for the brain examination. Therefore, PMI that can examine the intracerebral state of the cadaver is useful. The findings of PMI are interpreted by taking into consideration the postmortem changes based on the findings of imaging of the living body. Characteristic interpretations of the findings of PMI have yet to be developed.

In this paper, three cases of cerebral infarctions that were not found during life but were newly recognized on PMI are reported. The autopsies revealed severe systemic infectious diseases in all three cases. The aim of this paper is to suggest the possibility of the presence of a severe systemic infection when cerebral infarction is newly recognized on PMI.

## Case presentations

### Case 1

An 81-year-old woman visited a hospital for a pacemaker check. At that time, slightly impaired liver function was noted. She then developed a fever of 38 °C. Although careful examinations to identify the cause of the fever were performed, the source could not be identified. Various cultures were also negative. Although antibiotic treatment was given, her fever and general status did not improve, and she was admitted to our hospital. On physical examination at admission, her temperature was 38 °C, blood pressure was 140/87 mmHg, and her pulse was 76/min. On blood tests, hemoglobin (Hb) was 11.8 g/dl, C-reactive protein (CRP) was 5.54 mg/dl, soluble interleukin-2 receptor (sIL-2R) was 3732 U/ml (standard 144–518 U/ml), and a tendency to disseminated intravascular coagulation (DIC) (PLT 5.4 x 10^4^/μl, FDP 156 μg/ml, D-dimer 80 μg/ml) was seen. Slightly impaired liver function (aspartate aminotransferase (AST) 120 IU/l, alanine aminotransferase (ALT) 81 IU/l) was also found.

The chest CT on the second day after hospitalization showed a diffuse ground glass shadow and suspected interstitial pneumonia. On the fifth day after admission, contrast-enhanced CT showed a low-density area (LDA) in the left iliopsoas muscle, suggesting an iliopsoas muscle abscess. *Staphylococcus aureus*, *Escherichia coli (E. coli)*, or tuberculosis was considered as the causative organism of the iliopsoas muscle abscess. Various dysfunctions, such as interstitial pneumonitis, liver damage, and DIC, were also present simultaneously. We assumed that there was a solitary underlying disease that could explain her clinical picture, for example, hematological disease, and treatment was started.

Since intense accumulation of fluoro-deoxy-glucose (FDG) was observed in bilateral lung fields on FDG-positron-emission tomography (PET), an inflammatory disorder, especially tuberculosis was suspected, and three-agent combined therapy was started on the 8th day after admission. However, neither the fever nor her general status improved, and the ground glass appearance had deteriorated further on the chest CT on the 16th day after admission.

On the 22nd day after admission (9 days before death), the patient’s respiratory condition deteriorated suddenly, and methylprednisolone pulse therapy resulted in no improvement. She died on the 32nd day after hospitalization. PMI and an autopsy (only thoracoabdominal) were performed 14 h after death (Additional file 1).

#### PMI findings

**Fig. 1:**
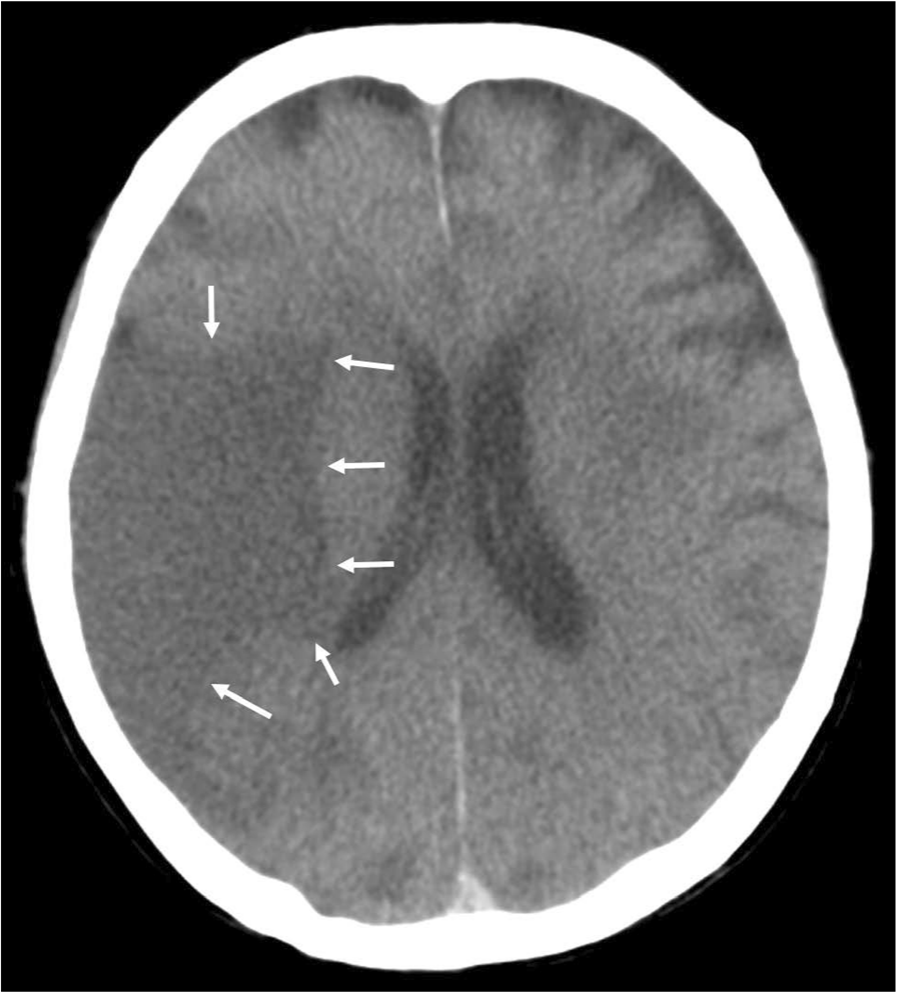
Brain postmortem CT image 14 h after death (Case 1). An LDA was found in the middle cerebral artery area (*arrows*)

Multiple LDAs were recognized in the right middle cerebral artery (MCA) region (Fig. 1), right cerebellum, and left basal ganglia on PMI. They seemed to be infarctions. Since no atrophy was found in the brain, the infarctions seemed to be relatively fresh lesions. There was neither a mass effect nor hemorrhage in the brain. The ground glass shadow was widespread in bilateral lungs, and part of the lungs showed infiltrative shadow and the crazy paving pattern. As the cause of the interstitial shadow, adult respiratory distress syndrome (ARDS), acute interstitial pneumonia, or an infectious disease such as *Pneumocystis jirovecii* pneumonia was considered. No airway obstruction was found. A cardiac pacemaker was confirmed. Neither brain CT nor brain MRI was done during the patient’s lifetime.

#### Pathological findings

**Fig. 2:**
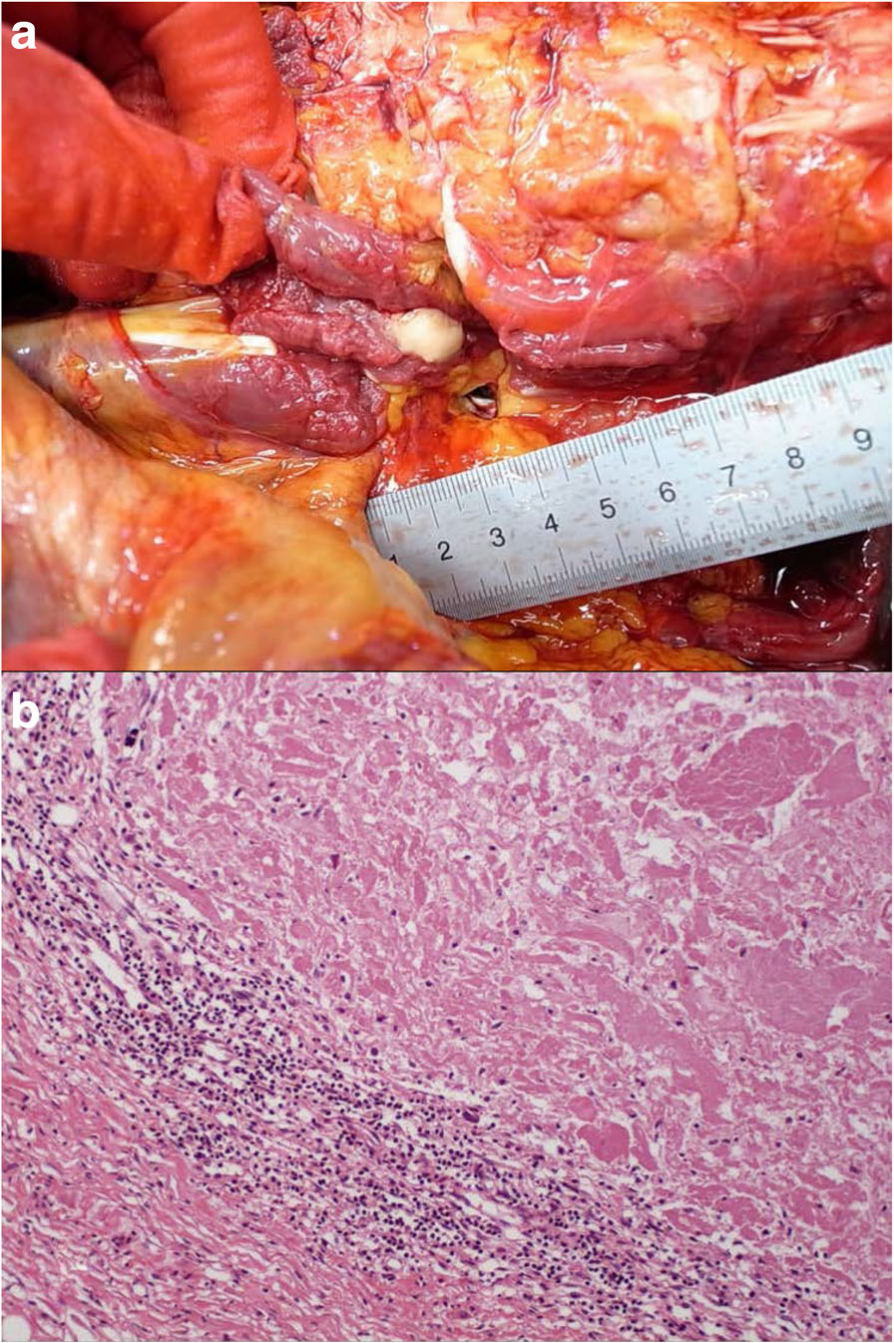
The cold abscess of the left iliopsoas muscle (Case 1). **a** The left iliopsoas muscle that was cut open at autopsy. White viscous liquid was seen. **b** The micrograph of the iliopsoas abscess. The content of the abscess is necrotic material, and neutrophilic infiltration is not seen (Hematoxylin-Eosin (HE) stain. Original magnification × 4)

**Fig. 3:**
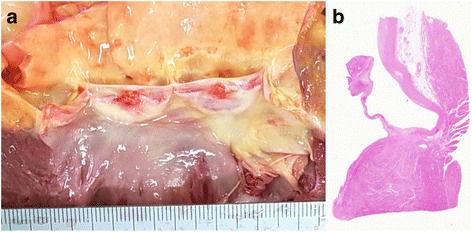
Vegetations of the aortic valve at autopsy (Case 1). **a** The macroscopic appearance of the aortic valve. The aortic valve has two vegetations of 4 mm and 5 mm in diameter. **b** Loupe image of the aortic valve. Vegetations consist of fibrin without bacterial colonies, and non-bacterial thrombotic endocarditis was diagnosed (HE stain. Original magnification × 1)

On autopsy, white viscous liquid flowed when an incision was made into the left iliopsoas muscle (Fig. 2a). The wall of the abscess was composed of lymphocytes and fibrous tissue, and the contents of the abscess consisted of necrotic material (Fig. 2b). A few neutrophils infiltrated into the abscess. A few acid fast bacilli were noted in the abscess with Ziehl-Neelsen staining. These histological findings and polymerase chain reaction (PCR) testing showed that the lesion was tuberculous. In addition, tuberculous nodules were found microscopically in bilateral lungs (left 730 g, right 842 g), liver (1118 g), spleen (94 g), left kidney (182 g), bone marrow, and lymph nodes surrounding the pancreas, and miliary tuberculosis was diagnosed. Moreover, vegetations (4 mm and 5 mm in diameter) were noted on the aortic valve (Fig. 3a). The vegetations consisted of fibrin thrombus without bacterial colonies (Fig. 3b), and they were diagnosed as non-bacterial thrombotic endocarditis. This thrombus might have detached from the valve and become the emboli that resulted in the cerebral infarctions.

### Case 2

A 74-year-old man was taking a steroid (30 mg/day of predonin) for systemic lupus erythematosus (SLE) and was being followed in the outpatient department. A blood test showed severe anemia (Hb 3.8 g/dl), and he was hospitalized 2 months before death. Melena was found, but no bleeding source was identified even on gastroscopy and colonoscopy. Then, 14 days after admission, free air was found at the subphrenic region on chest X-ray and CT, and intestinal perforation was suspected. The patient was given a course of antibiotic treatment and drainage of ascites because of his general status.

The patient’s manifestations were relieved, but the inflammatory response increased again, and his respiratory condition suddenly worsened, requiring intensive care unit (ICU) admission. The onset of ARDS was suspected. He had DIC on admission, and it was exacerbated with progression of the infection. His inflammatory response, renal failure, and respiratory condition deteriorated, and he died 2 months after admission. PMI and an autopsy (only thoracoabdominal) were performed 2 h after death (Additional file 2).

#### PMI findings

**Fig. 4:**
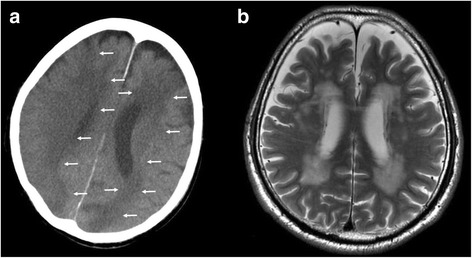
The postmortem CT and antemortem MRI (Case 2). **a** The postmortem CT image of the brain 2 h after death. LDAs were found widely, resulting in a diagnosis of cerebral infarction (*arrows*). (with permission [6]) **b** Antemortem MRI, T2-weighted image showing no cerebral infarction. The brain MRI was taken 1 year 5 months before death

Ascites and an abdominal aortic aneurysm were found on abdominal CT, but the free air had disappeared. On chest CT, infiltrative shadow was found, and ARDS, pneumonia, and interstitial pneumonia were considered. Brain CT showed a wide LDA area in the right parietal lobe, and cerebral infarction was diagnosed (Fig. 4a) [6]. Multiple new cerebral infarctions were seen on PMI. Brain MRI was done 1 year 5 months before death. The T2-weighted image of the MRI corresponding to the CT image is shown (Fig. 4b). At that time, no cerebral infarction was found.

#### Pathological findings

**Fig. 5:**
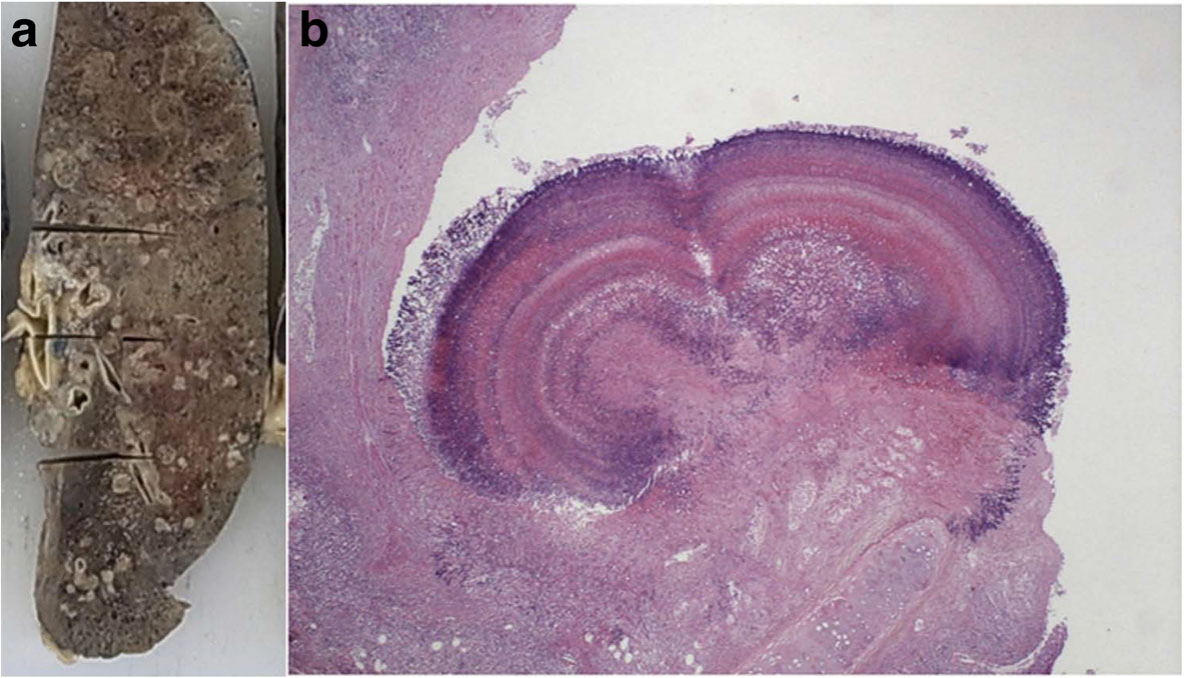
The aortic valve and mitral valve at autopsy (Case 2). **a** The macroscopic appearance of the aortic valve. The aortic valve has two vegetations of 4 mm and 5 mm. **b** The macroscopic appearance of the mitral valve. The mitral valve has some vegetations diagnosed as non-bacterial thrombotic endocarditis histologically. (with permission [6])

**Fig. 6:**
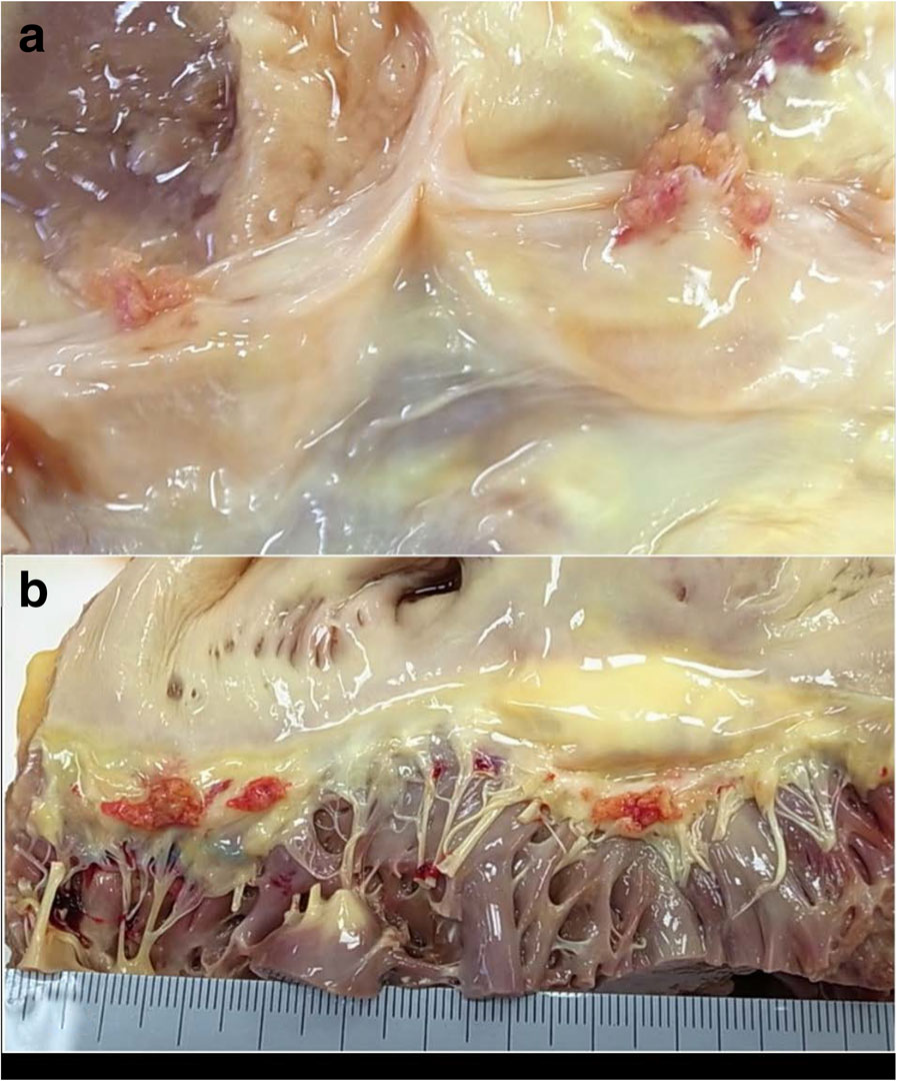
The lung after fixation (Case 2). **a** The macroscopic appearance of the lung. Diffuse small nodules were noted in the bronchi and parenchyma of the lung. **b** The microscopic appearance of the bronchus. The aspergillus had grown to project into the bronchus in the low-power image of the hilar region. (with permission [6])

Yellowish white purulent ascites of 1800 ml was found in the abdominal cavity at autopsy, and it showed diffuse peritonitis. However, the perforation site of the intestine was not confirmed. Moreover, invasive bronchopulmonary aspergillosis was present in the lungs (Fig. 5a,b) [6], and the background lung showed diffuse alveolar damage. Aspergillus species were also found in the peritoneum. There were a few vegetations, up to 10 mm in diameter, on the aortic valve in the heart (Fig. 6a) [6]. Three vegetations, 12 mm, 10 mm, and 7 mm in diameter, were also found on the mitral valve (Fig. 6b) [6]. They were regarded as the cause of the cerebral infarction; these thrombotic vegetations had separated from the valves. In addition, cholesterin crystal embolism was found in the kidney, heart, liver, spleen, and it was thought that this had caused the progressive renal dysfunction.

### Case 3

A 65-year-old man developed a left pontine infarction 2 months before hospitalization. He had a fever of about 39 °C. Blood tests showed white blood cells of 300/μl (3% neutrophils; neutropenia), and he was hospitalized in the Department of Hematology. Drug-induced neutropenia was suspected, and granulocyte colony-stimulating factor and antibiotics were administered with stopping of oral medicine. However, his fever remained, and pneumonia of both upper lobes was diagnosed by CT on the fourth day after admission, and an antifungal drug was added. Pain in both femoral regions then appeared. His pneumonia got worse, and intramuscular abscesses of both shoulders were noted 30 days before death. Though an antimicrobial drug was added, the pneumonia worsened, and an inflammatory pleural effusion developed. The patient’s level of consciousness decreased, and complete paralysis of the right side arm and leg appeared. He died 3 days after his consciousness level decreased and the right hemiplegia developed. PMI and autopsy were performed 2 h after death (Additional file 3).

#### PMI findings

**Fig. 7:**
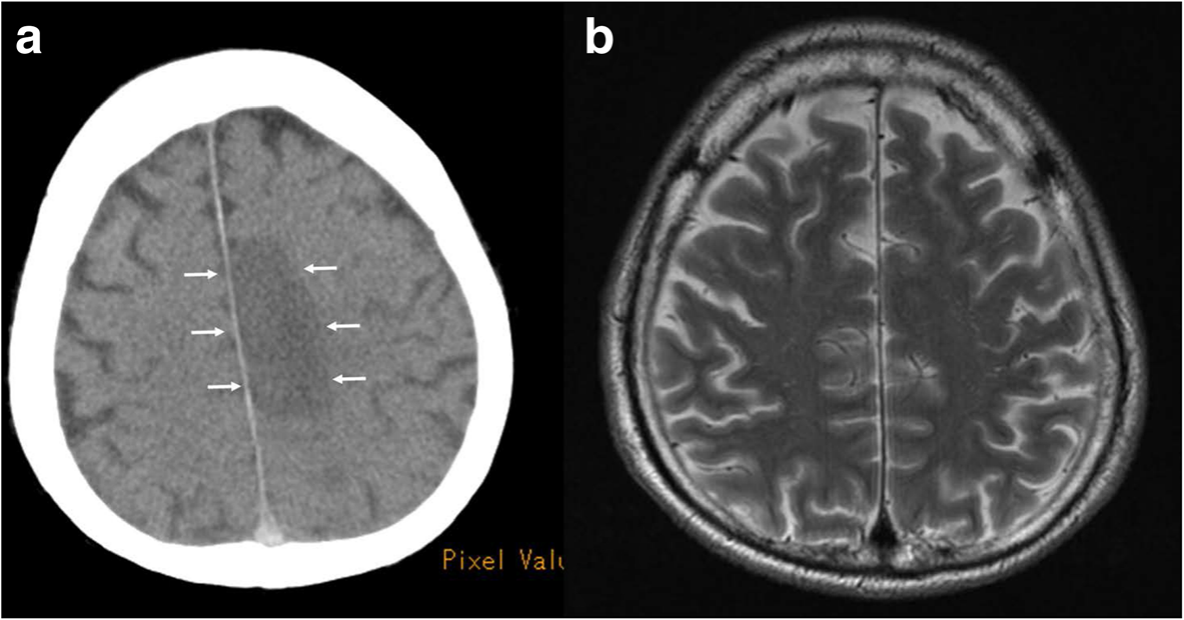
The postmortem CT and antemortem MRI (Case 3). **a** Brain postmortem CT image 7 h after death. The left parietal lobe has an LDA that was diagnosed as cerebral infarction (*arrows*). **b** Antemortem MRI, T2-weighted showing no cerebral infarction. The brain MRI was taken 22 days before death

An LDA was found in the left parietal lobe, and it was a newly diagnosed cerebral infarction on PMI (Fig. 7a). Brain MRI was done 22 days before death. The T2-weighted image of the MRI corresponding to the CT image is shown (Fig. 7b). At that time, no cerebral infarction was found.

#### Pathological findings

**Fig. 8:**
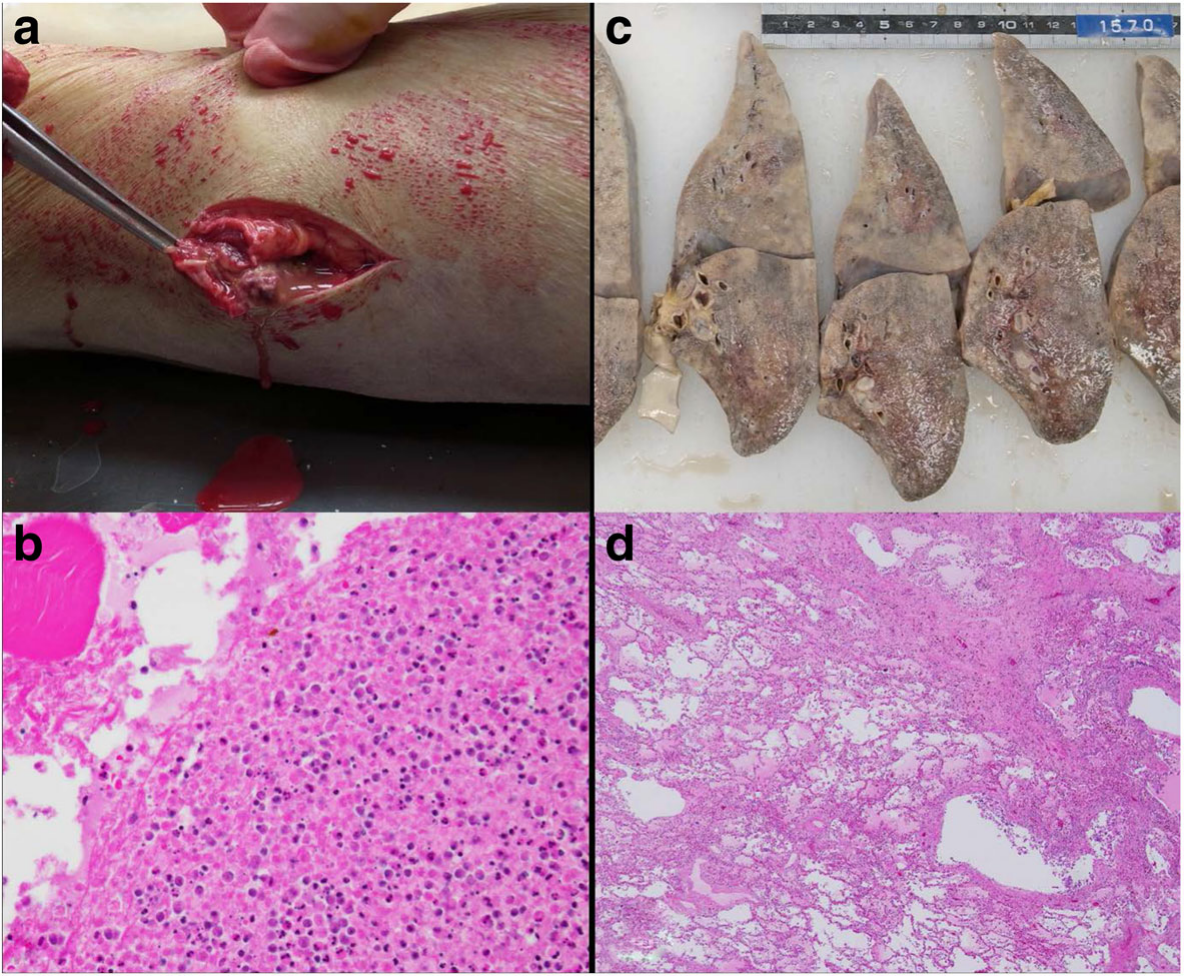
The left thigh and left lung at autopsy (Case 3). **a** The macroscopic appearance of the thigh. After incision into the abscess of the left thigh, leakage of pus is noted. **b** The microscopic appearance of the abscess. Numerous necrotic cells and neutrophils are noted. The pus was cultured, and *E. coli* was detected (HE stain. Original magnification × 20). **c** The cut surface of the left lung after fixation. The lung was diffusely firm, boggy, and heavy. Whitish lesions were found. **d** The microscopic appearance of the lung. The alveoli were filled with eosinophilic fluid and neutrophils (HE stain. Original magnification × 4)

The autopsy revealed a severe systemic infection: bilateral bronchopneumonia (left 954 g: right 857 g), right-sided pleuritis with effusion, and an intramuscular abscess in the right thigh (Fig. 8). Moreover, systemic fibrin thrombosis and a bleeding tendency due to DIC were seen. The thrombosis resulted in fresh multiple organ infarctions, such as myocardial infarction, left renal infarction, splenic infarction, and cerebral infarctions in the left frontal and parietal lobes. It was also confirmed that the old pontine infarction formed a cyst inside the left pons. The pus of the right thigh abscess was cultured at autopsy, and *E. coli* was detected. *E. coli* and *Burkholderia cepacia* were also detected by blood culture from the right atrium.

## Discussion

As described above, both cases 1 and 2 did not show neurological manifestations of the cerebral infarctions during their lifetime, and there were no findings on brain CT at the final antemortem imaging. However, in both cases, cerebral infarctions were recognized on the postmortem brain CT. In case 3, neurologic symptoms appeared in the agonal stage, and cerebral infarction was newly recognized on postmortem brain CT. The autopsy revealed severe infectious diseases in all three cases.

From October 2010 to January 2014, there were 106 cases that underwent both PMI and autopsy at the University of Fukui Hospital. PMI was performed at the Ai center of the University of Fukui with an 8-slice multi-detector CT scanner (Hitachi Medico, Tokyo, Japan) used exclusively for autopsies, as described previously [7]. The corpus was placed in the supine position, and a full-body scan from the vertex to the toes was performed. The scanning conditions were 120 kV, 250 mA, 8 × 2.5 collimation, 1.125 pitch, 0.8-s rotation time, 5-mm slice thickness, and 5-mm increments [7, 8]. No contrast reagent was used in these cases.

In three of 106 cases (2.8%), cerebral infarction was newly recognized on PMI, and severe infection was diagnosed on the subsequent autopsy. However, this rate is limited to our hospital, which is a limitation of this report. Many of the autopsied subjects may have had an increased frequency of severe infectious diseases such as ARDS, sepsis, and pneumonia. However, our recent autopsy-based study of histiocytic hyperplasia with hemophagocytosis (polyhemophagocytosis) [9] showed that the incidence of polyhemophagocytosis was equal to that shown in the German, US, and Japanese literature [9–11], suggesting that the characteristics of our autopsied subjects were not biased. Therefore, there might be such cases in other hospitals. However, the exact frequency of cerebral infarction being newly recognized on PMI and severe infection being diagnosed on autopsy is not known.

Some comparison studies of PMI findings and pathological diagnosis by autopsy have been done. In two UK centers in Manchester and Oxford, 182 unselected cases were assessed by radiology (CT and MRI) and autopsy for the cause of death [12]. In Aachen University Hospital, 29 cases were analyzed by CT and autopsy [13], and in the ICUs of Hamburg, 47 cases were analyzed by autopsy and PMI [14]. However, none of these reported cases similar to the ones presented here.

The reported case of a woman in her 40s with familial hypercholesterolemia is similar [15]. She underwent aortic valve replacement and coronary bypass surgery for aortic stenosis and stenosis of the left main coronary artery. However, she developed acute myocardial infarction and died of postoperative mediastinitis and multiple organ failure. PMI and autopsy were done. An LDA was found in the right parietal lobe on PMI. The autopsy revealed that she had disseminated cryptococcosis and developed multiple organ failure due to sepsis.

In the present three cases and the one case in the literature, the systemic infectious disease had already been known before death. However, if the cerebral infarction is newly found on PMI even if infection is not suspected or adequate testing is not possible before the death, systemic infectious diseases might be suspected.

The cause of cerebral infarction in all cases was considered to be a thrombus of thrombotic endocarditis or DIC due to the severe infection. In fact, some studies reported the association of cerebral infarction with prior infection and inflammatory processes [16–20]. However, no article has referred to the relationship between cerebral infarction in PMI and systemic infection. Thus, we would like to emphasize that findings of brain infarctions on PMI might imply systemic infection.

The rate of hospital autopsies has been decreasing worldwide in recent years. For example, autopsies are performed in less than 10% of all U.S. deaths [21]. In the United Kingdom, the mean hospital autopsy rate in 2013 was 0.69% of hospital deaths [22]. In Japan, according to the Japan Council for Quality Health Care, the autopsy rate in 2012 was 4.0%. To make matters worse, another consent is required for the brain examination, so the rate of brain examination at autopsy is still lower; it is only about 20% at our hospital. Therefore, the finding of brain infarction on PMI of the cadaver is important because it suggests the presence of systemic infection.

Systemic infection such as miliary tuberculosis is regarded as a disease for which diagnosis is difficult on PMI at present. However, there is a possibility that systemic infection might become a disease for which a diagnosis can be suspected on PMI based on an accumulation of cases similar to those presented here. Thus, we believe that we can improve the certainty of PMI by comparative examinations between PMI and autopsy findings.

## Conclusion

Three cases with systemic infectious disease were presented in this report. All of them showed newly recognized cerebral infarctions on PMI. Cerebral infarction that is newly recognized on PMI might suggest the presence of severe systemic infection.

## Additional files

Additional file 1: Timeline of Case 1. (DOCX 20 kb)
Additional file 2: Timeline of Case 2. (DOCX 19 kb)
Additional file 3: Timeline of Case 3. (DOCX 19 kb)

## Abbreviations

Ai: Autopsy imaging
ALT: Alanine aminotransferase
ARDS: Adult respiratory distress syndrome
AST: Aspartate aminotransferase
CRP: C-reactive protein
CT: Computed tomography
DIC: Disseminated intravascular coagulation
*E. coli*: *Escherichia coli*
FDG: Fluoro-deoxy-glucose
Hb: Hemoglobin
HE: Hematoxylin-eosin.
ICU: Intensive-care unit
LDA: Low density area
MCA: Middle cerebral artery
MRI: Magnetic resonance imaging
PET: Positron-emission tomography
PMI: Postmortem imaging
sIL-2R: Soluble interleukin-2 receptor
SLE: Systemic lupus erythematosus
