# Supplementary material for: Newly recognized cerebral infarctions on postmortem imaging: a report of three cases with systemic infectious disease
Source: BMC Med Imaging. 2017 Jan 10;17:4. doi: 10.1186/s12880-016-0174-4 (PMC5223344; doi:10.1186/s12880-016-0174-4)
Supplement: Additional file 1: — Timeline of Case 1. (DOCX 20 kb) [file 12880_2016_174_MOESM1_ESM.docx]

| Dates | Relevant Past Medical History and Interventions | | |
| --- | --- | --- | --- |
|  | An 81-year-old woman  Hypertension (70 years old), Pacemaker implantation due to complete atrioventricular block (80 years old) | | |
| Date | Summaries from Initial and Follow-up Visits | Diagnostic Testing  (including dates) | Interventions |
| About one month before  hospitalization | An 81-year-old woman visited a hospital for a pacemaker check. | Slightly impaired liver function  A fever of 38°C.  Although careful examinations to identify the cause of the fever were performed, the source could not be identified.  Various cultures were negative. | Antibiotic treatment |
| Hospitalization  (Day 0) | Her fever and general status did not improve, and she was admitted to our hospital. | On physical examination at admission; Body Temperature (BT): 38°C.  Blood pressure, 140/87 mmHg.  Pulse, 76/min.  White Blood Cell (WBC): 3.2 (X 10^9^/L)  Red Blood Cell (RBC): 4.02 (X 10^12^/L)  Hemoglobin (Hb), 11.8 g/dl,  Platelet (PLT): 54 (X 10^10^/L)  C-reactive protein (CRP), 5.54 mg/dl, soluble interleukin-2 receptor (sIL-2R), 3732 U/ml (standard 144-518 U/ml)  A tendency to DIC (PLT 5.4 x 104/μl, FDP 156 μg/ml, D-dimer 80 μg/ml)  Aspartate aminotransferase (AST), 120 IU/l.  Alanine aminotransferase (ALT) 81 IU/l |  |
| Day 2 |  | The chest CT showed a diffuse ground glass shadow and suspected interstitial pneumonia.  BT: 39.3  WBC: 4.4 (X 10^9^/L)  RBC: 3.81 (X 10^12^/L)  Hb: 11.0 (g/dL)  PLT: 54 (X 10^10^/L)  CRP: 8.07 (mg/dL) | Antibiotics were added. |
| Day 5 | We assumed that there was a solitary underlying disease that could explain her clinical picture, for example, hematological disease. | Contrast-enhanced CT showed a low-density area (LDA) in the left iliopsoas muscle, suggesting an iliopsoas muscle abscess.  (Staphylococcus aureus, Escherichia coli (E. coli), or tuberculosis was considered as the causative organism of the iliopsoas muscle abscess.)  BT: 38.1  WBC: 3.3 (X 10^9^/L)  RBC: 3.76 (X 10^12^/L)  Hb: 10.7 (g/dL)  PLT: 46 (X 10^10^/L)  CRP: 9.45 (mg/dL) | Antibiotics were added. |
| Day 16 | An inflammatory disorder, especially tuberculosis, was suspected, | Intense accumulation of fluoro-deoxy-glucose (FDG) was observed in bilateral lung fields on FDG-positron-emission tomography (PET).  BT: 37.4  WBC: 3.1 (X 10^9^/L)  RBC: 3.69 (X 10^12^/L)  Hb: 10.6 (g/dL)  PLT: 43 (X 10^10^/L)  CRP: 10.11 (mg/dL) | Three-agent combined therapy was started. |
|  | Neither the fever nor her general status improved, | The ground glass appearance had deteriorated further on the chest CT. |  |
| Day 22 | Respiratory condition suddenly deteriorated | BT: 37.5  WBC: 3.9 (X 10^9^/L)  RBC: 3.51 (X 10^12^/L)  Hb: 9.9 (g/dL)  PLT: 62 (X 10^10^/L)  CRP: 9.15 (mg/dL) | Methylprednisolone pulse therapy. (No improvement) |
| Day 31 |  | BT: 37.3  WBC: 7.4 (X 10^9^/L)  RBC: 3.68 (X 10^12^/L)  Hb: 10.2 (g/dL)  PLT: 70 (X 10^10^/L)  CRP: 10.17 (mg/dL) |  |
| Day 32 | She died. |  | PMI and an autopsy (thoracoabdominal) were performed 14 hours after death. |
