# Supplementary material for: Newly recognized cerebral infarctions on postmortem imaging: a report of three cases with systemic infectious disease
Source: BMC Med Imaging. 2017 Jan 10;17:4. doi: 10.1186/s12880-016-0174-4 (PMC5223344; doi:10.1186/s12880-016-0174-4)
Supplement: Additional file 2: — Timeline of Case 2. (DOCX 19 kb) [file 12880_2016_174_MOESM2_ESM.docx]

| Dates | Relevant Past Medical History and Interventions | | |
| --- | --- | --- | --- |
|  | A 74-year-old man  SLE, Steroid (30 mg/day of prednisolone) | | |
| Date | Summaries from Initial and Follow-up Visits | Diagnostic Testing  (including dates) | Interventions |
| Two months before death |  | A blood test showed severe anemia (Hb 3.8 g/dl) |  |
| Hospitalization  (Day 0) | Melena was found. | No bleeding source was identified on gastroscopy and colonoscopy.  Body temperature (BT):35.5 (deg C)  WBC: 4.3 (X 10^9^/L)  RBC: 1.10 (X 10^12^/L)  Hb: 3.8 (g/dL)  PLT: 37 (X 10^10^/L)  CRP: 3.03 (mg/dL) |  |
| Day 14 |  | Free air was found on chest X-ray and CT, and intestinal perforation was suspected.  BT: 38.1  WBC: 1.3  RBC: 2.76  Hb: 9.5  PLT: 37  CRP: 15.53 | Antibiotic treatment and drainage of ascites. |
|  | The patient’s manifestations were relieved | Inflammatory response increased again, and his respiratory condition suddenly worsened, requiring intensive care unit (ICU) admission.  The onset of ARDS was suspected |  |
| Day 34 | DIC was exacerbated with progression of the infection. | His inflammatory response, renal failure, and respiratory condition deteriorated.  BT: 37.1  WBC: 5.1  RBC: 2.51  Hb: 8.5  PLT: 46  CRP: 0.67 |  |
| Day 61 |  | BT: 36.5  WBC: 14.2  RBC: 2.77  Hb: 8.5  PLT: 14  CRP: 9.61 |  |
| Day 62 | He died. |  | PMI and an autopsy  (thoracoabdominal) were performed 2 hours after death. |
