# Supplementary material for: Newly recognized cerebral infarctions on postmortem imaging: a report of three cases with systemic infectious disease
Source: BMC Med Imaging. 2017 Jan 10;17:4. doi: 10.1186/s12880-016-0174-4 (PMC5223344; doi:10.1186/s12880-016-0174-4)
Supplement: Additional file 3: — Timeline of Case 3. (DOCX 19 kb) [file 12880_2016_174_MOESM3_ESM.docx]

| Dates | Relevant Past Medical History and Interventions | | |
| --- | --- | --- | --- |
|  | A 65-year-old man | | |
| Date | Summaries from Initial and Follow-up Visits | Diagnostic Testing  (including dates) | Interventions |
| Two months before hospitalization. | The left pontine infarction |  |  |
| Hospitalization  (Day 0) | Drug-induced neutropenia was suspected, | He had a fever of about 39°C. Blood tests showed white blood cells of 300/μl (3% neutrophils; neutropenia),  BT: 39.0  WBC: 0.3 (X 10^9^/L)  RBC: 3.83 (X 10^12^/L)  Hb: 12.0 (g/dL)  PLT: 216 (X 10^10^/L)  CRP: 19.38 (mg/dL) | Granulocyte colony-stimulating factor and antibiotics were administered with stopping of oral medicine. |
| Day 4 | His fever remained, | Pneumonia of both upper lobes was diagnosed by CT  BT: 37.2  WBC: 0.4 (X 10^9^/L)  RBC: 3.41 (X 10^12^/L)  Hb: 10.3 (g/dL)  PLT: 230 (X 10^10^/L)  CRP: 23.85 (mg/dL) | Antifungal drug was added. |
|  | Pain in both femoral regions then appeared. |  |  |
| Day 9 | His pneumonia got worse and intramuscular abscesses of both shoulders were noted | BT: 38.2  WBC: 24.0 (X 10^9^/L)  RBC: 3.11 (X 10^12^/L)  Hb: 9.6 (g/dL)  PLT: 235 (X 10^10^/L)  CRP: 11.47 (mg/dL) | Antimicrobial drug was added |
|  | The pneumonia worsened, and an inflammatory pleural effusion developed. |  |  |
| Day 36 | His consciousness level decreased and the right hemiplegia developed. | BT: 38.3  WBC: 24.7 (X 10^9^/L)  RBC: 3.24 (X 10^12^/L)  Hb: 9.6 (g/dL)  PLT: 134 (X 10^10^/L)  CRP: 15.62 (mg/dL) |  |
| Day 38 |  | BT: 37.5  WBC: 30.6 (X 10^9^/L)  RBC: 2.85 (X 10^12^/L)  Hb: 8.7 (g/dL)  PLT: 111 (X 10^10^/L)  CRP: 21.25 (mg/dL) |  |
| Day 39 | He died |  | PMI and autopsy were performed 2 hours after death. |
